# Synovial membrane protein expression differs between juvenile idiopathic arthritis subtypes in early disease

**DOI:** 10.1186/ar4434

**Published:** 2014-01-13

**Authors:** Sorcha Finnegan, Joanne Robson, Caitriona Scaife, Catherine McAllister, Stephen R Pennington, David S Gibson, Madeleine E Rooney

**Affiliations:** 1 Arthritis Research Group, Queen’s University Belfast, Centre for Infection and Immunity, Health Sciences Building, 97 Lisburn Road, Belfast BT9 7BL, UK; 2 School of Biological and Biomedical Sciences, Durham University, Durham DH1 3LE, UK; 3 Proteome Research Centre, UCD Conway Institute for Biomolecular and Biomedical Research, University College Dublin, Dublin Ireland; 4 Pediatric Rheumatology, Withers Ward, Musgrave Park Hospital, 20 Stockman's Lane, Belfast BT9 7JB, UK; 5 Northern Ireland Centre for Stratified Medicine, C-TRIC Building, Altnagelvin Hospital campus, Glenshane Road, Londonderry BT47 6SB, UK

## Abstract

**Introduction:**

Juvenile idiopathic arthritis (JIA) is the most common rheumatological disease of childhood with a prevalence of around 1 in 1,000. Without appropriate treatment it can have devastating consequences including permanent disability from joint destruction and growth deformities. Disease aetiology remains unknown. Investigation of disease pathology at the level of the synovial membrane is required if we want to begin to understand the disease at the molecular and biochemical level. The synovial membrane proteome from early disease-stage, treatment naive JIA patients was compared between polyarticular and oligoarticular subgroups.

**Methods:**

Protein was extracted from 15 newly diagnosed, treatment naive JIA synovial membrane biopsies and separated by two dimensional fluorescent difference in-gel electrophoresis. Proteins displaying a two-fold or greater change in expression levels between the two subgroups were identified by matrix assisted laser desorption ionization-time of flight mass spectrometry with expression further verified by Western blotting and immunohistochemistry.

**Results:**

Analysis of variance analysis (*P* ≤ 0.05) revealed 25 protein spots with a two-fold or greater difference in expression levels between polyarticular and oligoarticular patients. Hierarchical cluster analysis with Pearson ranked correlation revealed two distinctive clusters of proteins. Some of the proteins that were differentially expressed included: integrin alpha 2b (*P* = 0.04); fibrinogen D fragment (*P* = 0.005); collagen type VI (*P* = 0.03); fibrinogen gamma chain (*P* = 0.05) and peroxiredoxin 2 (*P* = 0.02). The identified proteins are involved in a number of different processes including platelet activation and the coagulation system.

**Conclusions:**

The data indicate distinct synovial membrane proteome profiles between JIA subgroups at an early stage in the disease process. The identified proteins also provide insight into differentially perturbed pathways which could influence pathological events at the joint level.

## Introduction

Juvenile idiopathic arthritis (JIA) consists of a heterogeneous group of diseases which persist for more than six weeks and commence before the age of 16. It is the most common rheumatological disease of childhood, with a prevalence of approximately 1/1,000 [1]. The cause of JIA remains unknown; however, environmental and genetic factors play a role in its pathogenesis and the heterogeneity of the disease would suggest multiple factors are responsible [2]. Adverse outcomes include chronic pain and stiffness, and joint damage and disability as a result of chronic joint inflammation. Despite improved prognoses for JIA patients in recent years, around half of patients will continue to have active joint disease into adulthood [3].

The International League of Associations for Rheumatology (ILAR) classification system divides JIA into seven clinical subgroups [4,5]. The largest subgroup - oligoarticular JIA - accounts for approximately 65% of all cases and refers to children with four or fewer joints involved within six months of diagnosis. Oligoarticular disease is further defined as persistent when it remains confined to four or fewer joints throughout the course of the disease. Oligoarticular JIA is more prevalent in females, with a peak age of onset of one to three years [6]. Polyarticular JIA is defined by the involvement of more than four joints within six months of diagnosis, it accounts for around 10 to 15% of all JIA cases [7].

Very few studies examine the synovial membrane from JIA patients and, of those available, most involved patients with established disease and on disease modifying treatment [8-11]. Our previous immunohistochemical study demonstrated significant differences in the inflammatory infiltrates and levels of vascularisation in the different JIA subgroups. It is still unclear, however, why these changes take place in JIA and why they differ between subgroups. Proteomics offers an unsupervised means to investigate the disease process within the affected tissue.

This study was designed to investigate how the predominant protein expression profile within synovial membrane varies between persistent oligoarticular and polyarticular JIA across a group of newly diagnosed, treatment-naïve patients. We hypothesize that subtype-specific protein expression patterns exist in the early stages of disease. Comparison of the synovial proteome between polyarticular and oligoarticular JIA patients could provide a much needed insight into the pathology underlying these two distinct subtypes.

## Materials and methods

### Patients

Children with newly diagnosed JIA of less than two years duration were consecutively recruited following informed consent. Medical Ethics Committee approval was obtained for this study at Green Park Healthcare Trust and patient assent and parent informed consent was given (ORECNI 408/03). Recruitment criteria included: involvement of one knee joint that required intra-articular steroid injection, and the patient being steroid and disease-modifying antirheumatic drug (DMARD) naive. Children were classified according to the ILAR criteria at the time of recruitment. Children were then reclassified after two years of follow-up. Detailed clinical examination, biochemical and immunological data, and imaging assessments were obtained. Subgroups were compared with each other as it was not ethically possible to obtain synovial membrane biopsies from healthy paediatric controls.

### Tissue collection

Synovial membrane samples were obtained by blind needle biopsy using ultrasound guidance as described previously [12]. Biopsies were obtained prior to the instillation of steroids. Tissue was wrapped in saline soaked gauze, immersed in saline and placed in sterile containers on ice. Biopsies were snap frozen in liquid nitrogen and stored at -80°C.

### Sample preparation

Protein was extracted from synovial membrane biopsies in lysis buffer (8.4 M urea, 2.4 M thiourea, 4% 3-[(3-cholamidopropyl)dimethylammonio]-1-propanesulfonate, 2 mM dithiothreitol (DTT), 1% ampholytes). Samples were centrifuged at 12,000 rpm for 15 minutes and the supernatant collected. Protein was precipitated in ice cold acetone at -20°C overnight, pelleted by centrifugation and the pellet air dried to remove residual acetone. The protein pellet was resuspended in difference in-gel electrophoresis (DIGE) lysis buffer (30 mM Tris-Cl, 7 M urea, 2 M thiourea, 4% CHAPS, pH 8.5) and protein concentration was determined using a modified Bradford assay.

### Difference in-gel electrophoresis (DIGE)

DIGE was performed as previously described [13] with the exception that isoelectric focusing (IEF) and 2D PAGE were performed on 11 cm Immobiline DryStrip pH 4 to 7 linear. Each gel contained one synovium sample from a polyarticular patient and one synovium sample from an oligoarticular patient (each labelled with either Cy3 or Cy5 dyes). Cy3 and Cy5 dyes were swapped randomly between samples to prevent dye bias. Each gel also contained the internal standard which is a pooled mixture of every patient sample (labelled with Cy2). Voltage and times were adjusted accordingly. Two preparative gels loaded with 300 μg of protein were Coomassie stained and spots excised for identification by mass spectrometry.

### Image analysis

DIGE gels were scanned using a Typhoon 9410 imager (GE Healthcare, Bucks, UK). Gel images were analysed using Progenesis Samespots software version 2.0 build 2644.18003 (Nonlinear Dynamics Ltd., Newcastle upon Tyne, UK) as previously described [14]. PermutMatrix [15] was subsequently used to cluster and visualise the spot data as previously described [14].

### Tryptic digestion of protein spots and MALDI target spotting

Tryptic digestion of protein bands was performed using a ProGest robot (Genomic Solutions, Holliston, MA, USA) programmed with the long trypsin digestion method. A sterile pipette tip, cut to increase the bore width (approximately 2.0 mm), was used for spot excision. Gel plugs were then transferred to a 96-well plate. This plate is designed with microscopic holes at the bottom of the wells which allows for positive displacement of liquids during reagent changes on the robot. Gel pieces were first equilibrated in 50 μl of 50 mM ammonium bicarbonate and proteins were reductively alkylated with 10 mM DTT and 100 mM iodacetamide followed by de-staining of the protein bands and desiccation of the gel plugs with acetonitrile. Gel plugs were rehydrated with 50 mM ammonium bicarbonate containing 5% trypsin and the proteins were digested for 12 hours at 37°C. Resulting peptides were extracted from the gel plugs with 2 × 25 μl washes of 50% acetonitrile, 0.1% TFA. Peptide extracts were freeze dried and then re-suspended in 10 μl of 0.1% formic acid. A saturated solution of α-cyano-4-hydroxy-cinnamic acid was prepared in 50% acetonitrile, 0.1%TFA, 10 mM ammonium acetate. For each sample, 1 μl of matrix solution was spotted on the matrix assisted laser desorption ionisation (MALDI) target immediately followed by 1 μl of sample into the matrix spot and the sample/matrix droplet allowed to slowly air dry.

### MALDI TOF-TOF analyses, database searching and protein identification

Matrix assisted laser desorption ionisation-tandem time of flight (MALDI TOF-TOF) analyses was performed on a 4800 mass spectrometer (AB Sciex UK Limited, Cheshire, UK) using the following protocol: TOF-MS analyses was first performed on all of the dried target spots using automated data acquisition and processing under the control of Applied Biosystems 4000 series Explorer software (version 3.5) using reflector mode, a mass range of 700 to 4,000 m/z, 1,000 total laser shots per spectrum and a laser intensity of 3,300 V. Following acquisition the TOF-MS spectra were noise corrected, peak de-isotoped and internally calibrated using the trypsin autolysis peaks 842.500 and 2,211.100 m/z. The eight most abundant precursor ions from each spectra were then selected by the software for fragmentation and MS-MS analyses using a 1 kV collision ion dissociation (CID) fragmentation method collecting 4,000 laser shots per spectra with a laser intensity of 3,800 over the mass range.

Peak lists of ion masses were generated by GPS Explorer software version 3.6 (Applied Biosystems) from the calibrated and de-isotoped MS and MS-MS spectra for each sample. Combined lists of MS and MS-MS data were used for database searching with MASCOT version 2.2 (Matrix Science), against all entries in the National Center for Biotechnology Information (NCBI) database. Database search parameters used were the following: digestion enzyme trypsin, single missed cleavage allowed, variable modifications of carboxymethyl cysteine and oxidised methionine, precursor mass tolerance of 50 ppm and fragment ion tolerance of 0.2 Da. HTML pages of MASCOT output were generated for each sample.

The Mowse scoring algorithm was used to determine the significance of the identity. Additional criteria were used to ensure correct protein identifications. These included ensuring the matched peptides were the most abundant peaks in the mass spectrum, the theoretical isoelectric point and molecular weight of the identified protein correlated with the spot’s position on the 2D gel, and sequence coverage was high.

### Immunohistochemistry

Biopsies were snap frozen in liquid nitrogen for sectioning and stored at -80°C. A total of 7 μm thick frozen sections were cut using a Leica CM 1900 cryostat (Leica Microsystems Nussloch GmbH, Germany) and allowed to air-dry at room temperature for 30 minutes. Primary antibodies used were monoclonal anti-integrin alpha 2b antibody (ITA2B) (Abcam, Cambridge, UK), monoclonal anti-peroxiredoxin 2 (PRDX2) (Sigma, Dorset, UK) and polyclonal anti-myosin regulatory light chain 2 (MYL2) (Sigma, Dorset, UK). Immunostaining was performed using a standard protocol and staining was visualized using the diaminobenzidine (DAB) method [16]. Sections were counterstained with haematoxylin.

### Western blotting

Western immunoblot analysis was performed using a standard protocol using the above antibodies against ITA2B, PRDX2 and MYL2.

## Results

### Patient demographics and clinical features

There were 12 females and 3 males included in the study. The clinical and laboratory data of patients are shown in Table 1.

**Table 1:** Demographic and clinical characteristics at the time of biopsy

**ILAR subtype**	**JIA patient study ID**	**M/F**	**Disease duration (months)**	**No. of swollen joints**	VAS* pain (parent)	VAS* global physician	WCC^*^	Hb^*^	ESR^*^	CRP* (mg/l)	PC^*^	ANA^*^	**RF (+/−)**
**Oligoarticular (n = 7)**	1	F	4	1	5	1.9	7.5	11	8	0	407	0	-
	2	M	4	1	0	0.8	12	11	11	0	415	0	-
	3	F	6	3	3.6	2.9	12.1	11	10	5.7	453	0	-
	4	F	10	2	0.3	1.9	6.9	10.7	4	4	315	0	-
	5	F	5	3	7.5	3	12.8	10.6	37	26	407	80	-
	6	M	20	1	1.4	1.9	5.6	14.1	5	0	249	0	-
	7	F	4	3	4.3	1.8	9.1	8.9	62	66.5	460	0	-
	**Mean values**		**7.6**	**2**	**3.2**	**2**	**9.4**	**11**	**19.6**	**14.6**	**386.6**	**11.4**	
**Polyarticular (n = 8)**	8	F	2	24	8.7	7.5	12.9	8.3	38	27.5	570	160	-
	9	F	2	9	8.3	3.3	13.9	10.4	22	36.1	232	0	-
	10	F	2	24	4.7	5.7	12.2	10.5	30	67.3	601	0	-
	11	F	2	6	9	4.8	9.9	11.1	26	32.1	446	80	-
	12	F	1	2	7.1	1.5	11.2	10.4	96	47.4	522	ND	-
	13	F	4	15	9.8	3.6	11.8	7.2	110	72	578	0	-
	14	M	10	4	3.3	5.3	10.27	11	20	5.4	314	40	+
	15	F	5	25	6.5	7.4	9.3	8.4	138	181	401	80	+
	**Mean values**		**3.5**	**13.6**	**7.2**	**4.9**	**11.4**	**9.7**	**60**	**58.6**	**458**	**51.4**	
	**t-test p value**		**ns**	**ns**	**0.004**	**0.002**	**0.056**	**0.049**	**0.029**	**0.03**	**0.12**	**-**	**-**

ANA, anti-nuclear antibodies; CRP, C-reactive protein; ESR, erythrocyte sedimentation rate; Hb, haemoglobin; ns, not statistically significant at *P* ≥0.05; PC, platelet count;; RF, rheumatoid factor; *VAS, visual analogue scale; WCC, white cell count.

### General laboratory characteristics of JIA patients

There was no significant difference in the age at disease onset between the two groups. VAS pain scores (parent) were significantly higher in the polyarticular as were ESR and CRP levels (Table 1).

### 2D DIGE analysis

**Figure 1:**
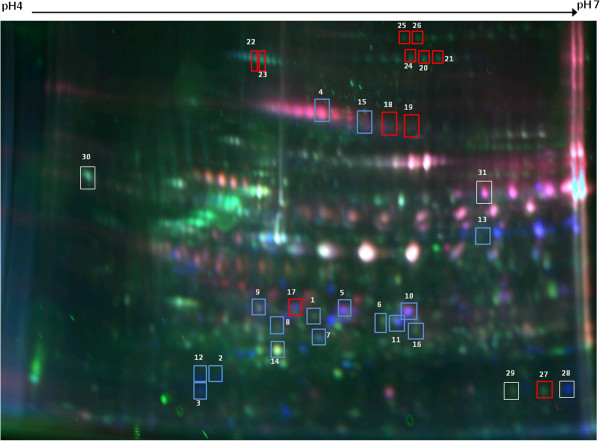
**A representative 2D DIGE gel image of JIA synovial membrane proteins.** Proteins labeled using fluorescent dyes Cy3 (green) and Cy5 (red). Protein samples were separated on an 11 cm pH4 to 7 IPG strip and on a 12.5% homogenous SDS-PAGE gel. Protein spots analysed by mass spectrometry are highlighted by spot outlines. Spot numbers correspond with those listed in Table 2, Additional file 1: Table S1 and Figure 2. Proteins overexpressed in oligoarticular patients are indicated by red spot outlines; proteins overexpressed in polyarticular are indicated by blue spot outlines. DIGE, difference in-gel electrophoresis; JIA, juvenile idiopathic arthritis.

We compared the synovial membrane proteome from seven persistent oligoarticular JIA patients with eight polyarticular JIA patients using 2D DIGE. 2D DIGE analysis revealed an average of 460 spots per pH 4 to 7, 11 cm gel which were matched across all gels (Figure 1). ANOVA revealed 26 spots that were differentially expressed (two-fold or more change in expression levels) between polyarticular and oligoarticular patients (*P* ≤0.05).

### Hierarchical cluster analysis (HCA)

**Figure 2:**
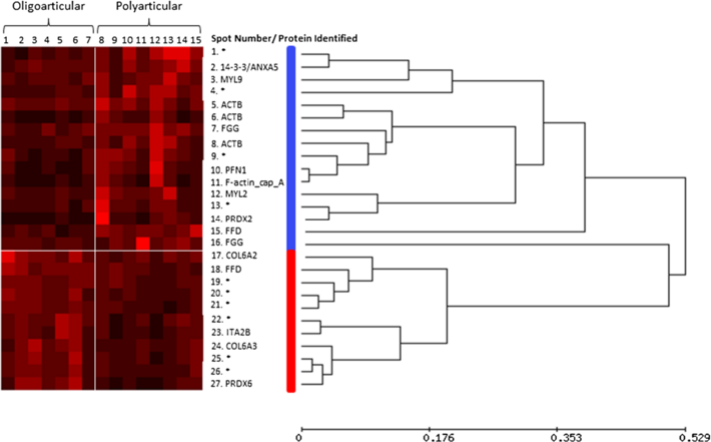
**Hierarchical cluster analysis of proteins expressed with statistically significant differences between patient subgroups.** The inter-individual and inter-group variation in 19 selected proteins is represented in the form of a heatmap. The protein expression data were reordered by hierarchical cluster analysis (HCA) using Pearson centred correlation (unweighted pair group method with arithmetic mean (UPGMA)), which revealed specific expression patterns. Two main clusters of proteins are highlighted by the coloured bars (blue, red). Each patient sample is represented by a single numbered column (oligarticular 1 to 7 and polyarticular 9 to 15). Spot numbers and protein identifications (UniProt code) are labelled on the vertical axis; unidentified protein spots are indicated by an asterix (*). The relative amount of a protein is denoted by colour intensity within a spectrum of black to red, red being the highest amount.

HCA was used to examine the expression patterns of the 26 differentially expressed proteins across the study cohort with results depicted in heat map form (Figure 2). Depicted on the horizontal axis is the patient number and on the vertical axis is the spot number/protein ID. Pearson ranked correlation revealed two distinct clusters of proteins. Cluster 1 contains proteins that were overexpressed in the polyarticular patients, whereas proteins in cluster 2 are overexpressed in the oligoarticular subgroup. It seemed conceivable that these two distinct clusters could be used in combination to differentiate these two disease subgroups.

### MALDI-TOF MS protein identification

Differentially expressed proteins were identified by MALDI MS (Table 2). Peptide ion validation data is available as an additional file (Additional file 1: Table S1). Proteins that were overexpressed in the polyarticular group included: chain A of profilin-beta-actin (*P* = 0.04); alpha-1 subunit of F-actin capping protein (*P* = 0.03); beta actin, (*P* = 0.04); beta actin (*P* = 0.02); myosin regulatory light chain 2 (MYL2) isoform B (*P* = 0.02); myosin regulatory light chain 9 (MYL9), isoform a (*P* = 0.04); beta actin variant (*P* = 0.005); peroxiredoxin 2 (PRDX2), isoform a (*P* = 0.02); mixture of protein kinase C inhibitor protein 1 (KCIP-1) and annexin A5 (*P* = 0.003); fibrinogen gamma chain (*P* = 0.004 and *P* = 0.05).

**Table 2:** Mass spectrometry and DIGE quantification data of synovial membrane proteins

**Spot number**	**Protein**	**Gene symbol**	**Accession #**	**Mowse score**	**% Sequence coverage**	**CalculatedpI value**	**Nominal mass (Mr)**	**Number matched peptides**	**Normalised volume (Oligo)**	**Normalised volume (Poly)**	**ANOVA** ***P*-value**
1	No ID	-	-	-	-	-	-	-	0.73	3.02	0.001
2	Protein kinase C inhibitor protein 1	*KCIP-1*	gi|114629705	88	40	4.78	24,166	14	1.07	2.1	0.03
	Annexin	*A5 ANXA5*	gi|149698420	79	33	4.98	35,977	10	-	-	-
3	Myosin regulatory l ight chain 9	*MYL9*	gi|29568111	52	29	4.8	19,814	8	0.92	2.02	0.04
4	No ID	-	-	-	-	-	-	-	0.79	2.35	0.006
5	Beta Actin	*ACTB*	gi|15277503	76	21	5.55	40,194	9	1.14	2.28	0.04
6	Beta Actin	*ACTB*	gi|15277503	176	37	5.55	40,194	17	0.35	1.24	0.02
7	Fibrinogen gamma chain	*FGG*	gi|930064	94	50	6.49	24,109	11	0.88	2.23	0.004
8	Beta actin variant	*ACTB*	gi|62897625	451	53	5.37	41,738	23	0.56	1.94	0.005
9	No ID	-	-	-	-	-	-	-	0.64	2.04	0.02
10	Chain A, profi l in-beta-actin	*PFN1*	gi|157111829	106/130	44	5.29	41,616	21	0.39	1.76	0.04
11	F-actin capping protein alpha-1	*CAPZA1*	gi|5453597	196	55	5.45	32,902	17	0.3	1.38	0.03
12	Myosin regulatory l ight chain 2	*MYL2*	gi|222144328	86	55	4.26	17,745	10	0.66	2.03	0.02
13	No ID	-	-	-	-	-	-	-	0.62	1.72	0.05
14	Peroxiredoxin	*2 PRDX2*	gi|4758638	348	57	6	25,019	18	0.3	1.73	0.02
15	Chain C, fibrinogen fragment D	*FFD*	gi|2781209	89	42	5.87	36,157	12	2.08	0.58	0.0002
16	Fibrinogen gamma chain	*FGG*	gi|930064	71	44	6.49	24,109	7	0.9	2.14	0.05
17	Collagen, type VI, alpha 2	*COL6A2*	gi|41350923	81	18	5.85	182,860	18	2.77	1.09	0.03
18	Chain C, fibrinogen fragment	*D FFD*	gi|24987625	102	44	5.86	35,155	14	2.1	0.96	0.005
19	No ID	-	-	-	-	-	-	-	1.6	0.9	0.008
20	No ID	-	-	-	-	-	-	-	1.6	0.8	0.02
21	No ID	-	-	-	-	-	-	-	1.4	0.7	0.006
22	No ID	-	-	-	-	-	-	-			
23	Integrin, alpha 2b	*ITGA2B*	gi|119571979	95	15	5.41	103,127	16	1.87	0.64	0.04
24	Alpha 3 type VI collagen	*COL6A3*	gi|62088852	119	17	8.73	182,860	27	1.78	0.78	0.05
25	No ID	*-*	-	-	-	-	-	-	1.72	0.74	0.04
26	No ID	*-*	-	-	-	-	-	-	1.56	0.72	0.02
27	Peroxiredoxin 6	*PRDX6*	gi|4758638	348	57	6	25,019	18	-	-	-
28	Triosephosphate isomerase 1	*TPI1*	gi|17389815	111	53	6.45	26,625	15	-	-	-
29	Albumin, isoform CRA_j	*ALB*	gi|119626073	59	23	6.4	26,233	7	-	-	-
30	Calreticul in precursor variant	*CRT*	gi|62897681	236	29	4.3	46,890	19	-	-	-
31	Fibrinogen, beta chain	*FGB*	gi|70906435	93	34	8.54	55,892	21	-	-	-

Spot trypsin digests were identified using matrix assisted laser desorption ionization (MALDI-TOF/TOF), correlated to compiled peptide data (Matrix Science). *P*-values highlight inter-subgroup comparisons which reached statistical significance (*P* <0.05) by ANOVA. Full mass spectra peak lists for each identified protein are provided in Additional file 1: Table S1.

**Figure 3:**
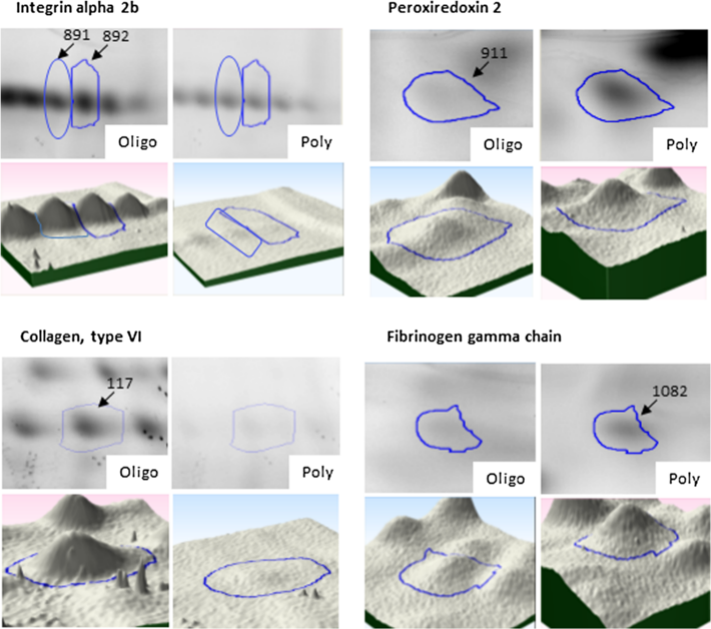
**DIGE image analysis of differentially expressed synovial membrane proteins.** Spots 22, 23 (integrin alpha 2b) and 24 (collagen) were overexpressed in the oligoarticular group. Spots 14 (peroxiredoxin 2) and 16 (fibrinogen gamma chain) were overexpressed in the polyarticular group. JIA, juvenile idiopathic arthritis.

Proteins overexpressed in the oligoarticular group included: collagen type VI, alpha 2 (*P* = 0.03); integrin alpha 2b (ITA2B) (*P* = 0.04); chain C of fibrinogen fragment D (*P* = 0.005); Chain C, crystal structure of fibrinogen fragment D (*P* = 0.0002). Figure 3 shows representative differentially expressed proteins along with a 3D image of the relative quantities of proteins generated using Samespots software (Nonlinear Dynamics Ltd., Newcastle upon Tyne, UK).

The levels of proteins 27 to 31 (listed in Table 2) did not differ between oligoarticular and polyarticular patient groups. There were a number of proteins that could not be identified due to their low abundance in the gel.

### Confirmation of protein localisation and expression

**Figure 4:**
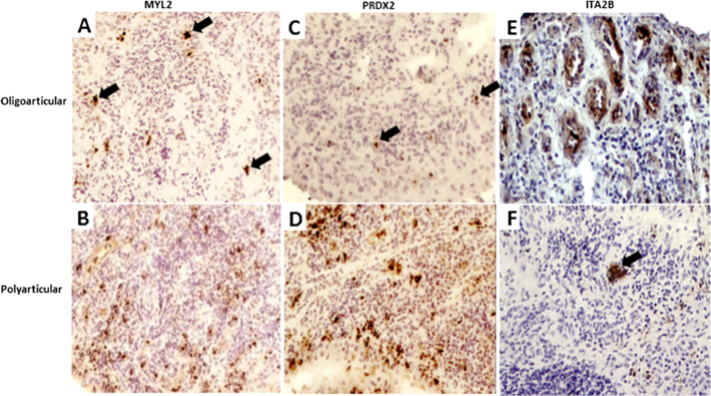
**Immunohistochemical staining of differentially expressed synovial membrane proteins. A** and **B** Distribution of myosin regulatory light chain 2 (MYL2), **C** and **D** peroxiredoxin 2 (PRDX2), **E** and **F** integrin alpha 2b (ITA2B) in oligoarticular and polyarticular synovial membrane. Sections were counterstained with haematoxylin. Arrows indicate areas of positive staining.

**Figure 5:**
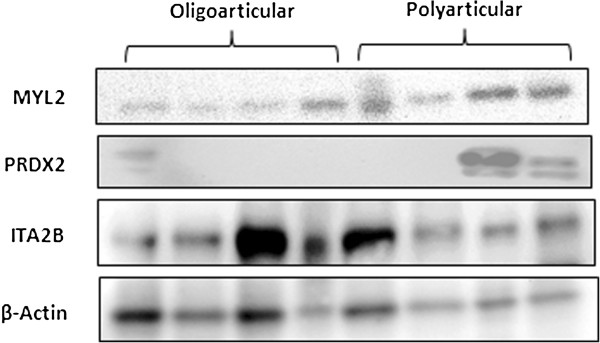
**Validation of protein expression changes by Western immunoblot probing for MYL2, PRDX2 and ITA2B.** Each lane represents an individual patient synovial membrane protein sample. β-actin was used as a loading control. ITA2B, integrin alpha 2b; MYL2, myosin regulatory light chain 2; PRDX2, peroxiredoxin 2.

Figure 4 shows representative images of MYL2, PRDX2 and ITA2B immunostaining in polyarticular and oligoarticular synovial membrane: (A) MYL2 staining is limited to a small number of discrete cells within the sublining layer in the oligoarticular tissue (arrows), its expression in the polyarticular tissue (B) is more intense. PRDX2 staining is sparse in the oligoarticular tissue (arrows) (C) with more intense staining in the polyarticular tissue (D). ITA2B staining is localised to blood vessels in oligoarticular tissue (E), some ITA2B staining was also observed in the polyarticular tissue (F) around blood vessels (arrows). Figure 5 depicts the synovial membrane expression levels for the aforementioned proteins across a representative subset of the study patients.

## Discussion

This is the first study to identify synovial membrane protein expression patterns that discriminate clinical subgroups in early treatment of naive JIA. By understanding the early pathological changes taking place at the disease site we can begin to elucidate the disease process. There have been a number of proteomic investigations of synovial tissue in adult inflammatory disease [17-19] but not in children. To date, proteomic studies of JIA have focused on synovial fluid and plasma [13,14,20-22], due to the fact that synovial membrane is extremely difficult to acquire. The synovial membrane has an advantage over synovial fluid in that it is the actual site of pathology in JIA. It is also important to note that all synovial membranes used in this study were from treatment-naive early disease-stage patients (average disease duration at time of biopsy was 5.4 months). For the purpose of this study the differences in two JIA subgroups were noted. Although control tissue from healthy subjects would have been useful, such tissue is not obtainable for ethical reasons.

A number of proteins identified in this study were part of a ‘charge train’ on the 2D gels, indicating post translational modification (PTM). A recently published study by our group indicates that protein modification acts as a surrogate marker of inflammation spread in JIA [23]. Glycosylation of VDBP- Vitamin D-binding protein, which confers macrophage activation activity, was decreased in patients at risk of disease spread. Subtype-specific protein isoforms uncovered within the current study should also be carefully investigated for post translational modifications. In early disease discrete protein isoforms may play a role in the different pathologies observed between oligoarticular and polyarticular synovium. In an earlier study we reported histological features which distinguish these JIA subgroups, including levels of vascularisation and white blood cell infiltration [16]. The likely pathological impact of synovial protein isoforms identified in the current study are subsequently discussed, along with their potential as therapeutic targets.

ITA2B was overexpressed in the oligoarticular group as part of a ‘charge train’ of spots (Figure 3), strongly suggesting a process of serial post translational modifications. ITA2B contains five N-linked glycosylation points [24]. Furthermore, intense ITA2B immunostaining was observed around blood vessels in the oligoarticular group, relative to polyarticular tissue. ITA2B is a major integrin found on platelet plasma membranes, assisting platelet activation and adhesion and thrombus formation at sites of vascular injury [25]. A previous study reported similar ITA2B expression patterns in rheumatoid arthritis (RA) synovial membrane, restricted to vascular endothelial cells [26]. ITA2B could act as a key target in controlling inflammation, as platelets can elicit cytokine responses from synovial fibroblasts via interleukin-1 in RA patients. The apparent increase in ITA2B observed in oligoarticular patients may result from leaky and tortuous vessels observed in the development of synovitis.

Fibrinogen fragment D was also expressed at higher levels in the oligoarticular group as part of a ‘charge train’. The D-dimer of fibrin is a degradation product (FDP) found in the blood following fibrinolysis of a blood clot [27]. Under various inflammatory conditions, including RA, fibrin degradation product levels are increased [28,29], suggesting an imbalance in the coagulation/fibrinolysis system. D-dimers have also been shown to trigger release of IL-1β, IL-6 in peripheral blood monocytes [30]. Fibrinogen is one of several protein targets for citrullination in RA [31] and citrullinated fibrinogen D fragments have been detected in synovial exosomes from patients with RA and osteoarthritis (OA).

In contrast, fibrinogen beta and gamma chains were overexpressed in the polyarticular group. Fibrinogen gamma chains are involved in fibrin polymerization and cross-linking, interacting with platelets and mediating thrombin binding to fibrin [32]. Rosenkranz *et al*. also observed that fibrinogen gamma chain levels were increased in systemic JIA synovial fluid relative to oligoarticular patients [21]. Fibrinogen beta chain is also increased in patients with recurrent joint inflammation [22]. A recent study also demonstrated strong reactivity of JIA sera to anti-citrullinated fibrinogen antibodies [33]. Fibrinogen has been shown to be the target of citrullination not only in JIA but also in adult RA [34], suggesting it may have an important role to play in autoimmunity.

During a normal inflammatory response, activation of coagulation and deposition of fibrin are crucial events that help regulate and contain inflammatory activity to the site of injury or infection. It is, therefore, possible that altered levels of coagulation system proteins reported in polyarticular tissue may indirectly assist in the spread of inflammation to multiple joints, a key characteristic of these patients. It would be intriguing to target these fragments in an animal model of arthritis to determine their function in the synovium.

The collagens are a superfamily of extracellular matrix proteins that play a crucial role in maintaining tissue integrity. Type VI collagen is a prominent constituent of the synovial extracellular matrix [35]. A study by Alexopoulos *et al.* suggests that type VI collagen has an important role in the regulation of normal synovial joint physiology and pericellular matrix displayed significantly reduced mechanical properties in mice lacking type IV collagen. Thus, as the mice aged, accelerated development of joint degeneration, as well as a number of other musculoskeletal abnormalities, was noted [36]. Collagen type VI was found at higher levels in the oligoarticular subgroup. Disease pathology is much less severe in this group, and it is possible that this is due to increased tissue integrity and a more stable extracellular and pericellular matrix in the synovial membrane.

A number of actin family proteins were overexpressed in the polyarticular group. Citrullinated F-actin capping protein alpha-1 subunit autoantigens have been identified in RA synovium [19]. It is thought that one of the pathogenic mechanisms taking place in RA is the promotion of actin polymerization and rearrangement of the actin cytoskeleton. Indeed, a previous study found a number of deregulated genes in synovial fibroblasts known to be involved in actin filament and cytoskeleton organisation [37]. Reorganisation of the actin cytoskeleton and the associated deregulation of ECM adhesion are known to be an intrinsic property of arthritic synovial fibroblasts. The presence of higher levels of these cytoskeletal proteins in the polyarticular group could reflect the severe disease pathology in this group and is consistent with much higher levels of synovial hyperplasia and cytoskeletal reorganisation.

PRDX2 was found at higher levels in the polyarticular group. Peroxiredoxin is involved in redox regulation. Peroxiredoxins constitute a ubiquitous family of antioxidant enzymes that are involved in the control of cytokine-induced peroxide levels [38]. Lymphocytes from RA patients have been shown to have increased levels of intracellular PRDX2 when compared to healthy controls [39]. Autoantibodies against peroxiredoxin have also been identified in RA [40]. Increased levels of PRDX2 in the polyarticular patients may indicate a dysregulated redox response system similar to that observed in adult RA.

## Conclusions

There are still substantial gaps in our understanding of JIA disease pathogenesis. As a result, JIA specific treatments have lagged behind the management of other adult arthritides. Identifying pathology specific proteins with functions in key cellular processes may provide help to prioritize putative therapeutic targets. We hypothesize that an imbalance in clotting factors, which primarily control plasma cell activation and thrombus formation, is associated with the involvement of multiple synovial joints in early stage JIA. In the case of polyarticular patients, diminished levels of specific clotting factors may prevent the repair of leaky neovasculature and increase the chances that multiple joints will be affected. Further targeted investigations of the circulating levels of the aforementioned factors in a suitably powered independent study cohort are required to robustly test this theory. Murine knockout models of arthritis which can sequentially test the function of the identified members of clotting pathways could also be used to verify this hypothesis.

Alternately, if the integrin alpha 2b is expressed exclusively on pathogenic vasculature, this property could be exploited to specifically target poorly formed vessels with integrin recognition (RGD) peptides. Integrin homing-RGD peptides have been shown to preferentially target inflamed synovium vessels without damaging other synovial tissues [41]. These small proteins could be linked to therapeutic agents, or clotting agents to clog the vessels which fuel the ensuing synovitis.

In summary, proteomic analysis of synovial tissue from early disease-stage JIA patients has revealed novel pathological protein expression patterns. With further work, molecular pathways which discriminate JIA subtypes could be exploited to develop new treatment strategies which suppress disease and reduce the risk of long term joint damage.

## Abbreviations

2D DIGE: Two dimensional fluorescent difference in-gel electrophoresis; 2D PAGE: Two dimensional polyacrylamide gel electrophoresis; ANOVA: Analysis of variance analysis; CHAPS: 3-((3-Cholamidopropyl) dimethylammonio)-1-propanesulfonate; CID: Collision induced dissociation; CRP: C-reactive protein; Da: Dalton; DAB: 3,3'-Diaminobenzidine; DMARD: Disease modifying anti-rheumatic drug; DTT: Dithiothreitol; ECM: Extracellular matrix; ESR: Erythrocyte sedimentation rate; FDP: Fibrin degradation product; HCA: Hierarchical cluster analysis; HTML: Hypertext markup language; IEF: Isoelectric focusing; IL-1β/6: Interleukin 1 beta or 6; ILAR: International league of associations for rheumatology; ITA2B: Integrin alpha 2b; JIA: Juvenile Idiopathic Arthritis; KCIP-1: Protein kinase C inhibitor protein 1; kV: Kilovolt; m/z: Ion mass over charge; MALDI-TOF: Matrix assisted laser desorption ionization-time of flight; MS-MS: Tandem mass spectrometry; MYL2/9: Myosin regulatory light chain 2 or 9; NCBI: National center for biotechnology information; OA: Osteoarthritis; ppm: Parts per million; PRDX2: Periredoxin 2; PTM: Post-translational modification; RA: Rheumatoid arthritis; RGD: Arginine-glycine-aspartate integrin recognition sequence; rpm: Revolutions per minute; TFA: Trifluoroacetic acid; TOF-MS: Time of flight mass spectra; UPGMA: Unweighted pair group method with arithmetic mean; VAS: Visual analogue scale.

## Competing interests

The authors declare they have no competing interests.

## Authors' contributions

SF and CS carried out the DIGE analysis. JLR performed the mass spectrometry. SF conducted the Western blot analyses and immunohistochemistry. CMcA collated all the patient demographic and clinical data. MR obtained consent from patients and collected tissue biopsies. DSG conceived the study and, along with SRP and MR, participated in its design and coordination. SF, MR and DSG wrote the manuscript. All authors read and approved the final manuscript.

## Supplementary Material

Additional file 1: Table S1 Peptide ion validation data from collision ion dissociation (CID) MS/MS mass spectrometry which correspond with Table 2. Peptide position in the matched protein, observed mass (m/z) and amino acid sequence are listed for each protein match found. Click here for file
